# Mapping the Universe of Eph Receptor and Ephrin Ligand Transcripts in Epithelial and Fiber Cells of the Eye Lens

**DOI:** 10.3390/cells11203291

**Published:** 2022-10-19

**Authors:** Michael P. Vu, Catherine Cheng

**Affiliations:** School of Optometry and Vision Science Program, Indiana University, Bloomington, IN 47405, USA

**Keywords:** RT-PCR, EphA2, ephrin-A5, knockout, variants, Eph-ephrin, bidirectional signaling

## Abstract

The eye lens is a transparent, ellipsoid organ in the anterior chamber of the eye that is required for fine focusing of light onto the retina to transmit a clear image. Cataracts, defined as any opacity in the lens, remains the leading cause of blindness in the world. Recent studies in humans and mice indicate that Eph–ephrin bidirectional signaling is important for maintaining lens transparency. Specifically, mutations and polymorphisms in the EphA2 receptor and the ephrin-A5 ligand have been linked to congenital and age-related cataracts. It is unclear what other variants of Ephs and ephrins are expressed in the lens or whether there is preferential expression in epithelial vs. fiber cells. We performed a detailed analysis of Eph receptor and ephrin ligand mRNA transcripts in whole mouse lenses, epithelial cell fractions, and fiber cell fractions using a new RNA isolation method. We compared control samples with EphA2 knockout (KO) and ephrin-A5 KO samples. Our results revealed the presence of transcripts for 12 out of 14 Eph receptors and 8 out of 8 ephrin ligands in various fractions of lens cells. Using specific primer sets, RT-PCR, and sequencing, we verified the variant of each gene that is expressed, and we found two epithelial-cell-specific genes. Surprisingly, we also identified one Eph receptor variant that is expressed in KO lens fibers but is absent from control lens fibers. We also identified one low expression ephrin variant that is only expressed in ephrin-A5 control samples. These results indicate that the lens expresses almost all Ephs and ephrins, and there may be many receptor–ligand pairs that play a role in lens homeostasis.

## 1. Introduction

In the anterior chamber of the eye, the lens, an ellipsoid and transparent tissue, is responsible for the fine focusing of light onto the retina to transmit a clear image. The function of the lens depends on its shape, biomechanical properties, clarity, and refractive index [1]. Despite decades of study, cataracts, defined as any opacity in the normally clear lens, remain the leading cause of blindness in the world [2]. The causes for congenital cataracts due to genetic mutations have been studied, but the cellular and molecular mechanisms that lead to age-related cataracts remain unclear.

Recent studies have shown that the dysfunction of Eph–ephrin bidirectional signaling leads to congenital and age-related cataracts in human patients [3,4,5,6,7,8,9,10,11,12,13,14]. Erythropoietin-producing hepatocellular carcinoma (Eph) receptors are the largest class of receptor tyrosine kinases (RTK). Eph receptors bind to a class of ligands, known as ephrins, and the binding of the receptor and ligand leads to a unique bidirectional signaling pathway with forward signaling in the Eph-bearing cell and reverse signaling in the ephrin-bearing cell [15,16,17]. Eph–ephrin bidirectional signaling is important for many cellular functions, including cell migration, proliferation, adhesion, and patterning [18,19,20,21]. Virtually all cells express a complement of Ephs and ephrins. There are 14 Eph receptors that are divided into EphAs (9 members, 1–8 and 10) and EphBs (5 members, 1–4 and 6), based on their sequence similarity and ligand affinity [17,22,23,24,25,26]. Ephrin ligands are categorized by structure into ephrin-As (5 members, 1–5), which are anchored via a glycosylphosphatidylinositol (GPI) moiety to the membrane, and ephrin-Bs (3 members, 1–3), which have a transmembrane region and a short cytoplasmic tail [17,24,25,27]. In human patients, mutations of the *EPHA2* gene cause congenital [3,4,5,6,7,8,9] and age-related [10,11,12,13] cataracts, while polymorphisms of the *EFNA5* gene, which encodes the ephrin-A5 protein, are linked to age-related cataracts [11].

The lens is composed of two cell types, a monolayer of epithelial cells covering the anterior hemisphere and a bulk mass of elongated and differentiated fiber cells [1]. The lens capsule is a basement membrane that surrounds the entire tissue, and lens epithelial cells are strongly adhered to the lens capsule [1]. Anterior epithelial cells are quiescent and thought to nourish the fiber cell underneath, and epithelial cells at the lens equator proliferate, migrate, elongate, and differentiate into new layers of lens fiber cells [28,29,30]. The addition of fiber cell layers along the lens equator drives life-long lens growth. Studies in mouse models indicate that EphA2 is required for hexagonal packing, for the organization of equatorial lens epithelial cells [31,32,33], and for fiber cell maturation [33,34,35], while ephrin-A5 is needed for maintaining the quiescence of anterior epithelial cells [32,36]. Recent works reveal that EphA2 and ephrin-A5 affect lens fiber cell patterning [33,34,37], which alters tissue biomechanical properties [34]. Immunostaining studies indicate that ephrin-A5 is mainly in the anterior epithelial cells, anterior tips of fiber cells, and peripheral equatorial fibers in mouse lenses [32,33,36]. In contrast, EphA2 proteins are mainly found along the equatorial epithelial cell and fiber cell membranes and in anterior fiber cell tips [10,32,33,36]. From these studies, it is possible that EphA2 and ephrin-A5 are a receptor–ligand pair at the anterior tips of lens fiber cells [33,34]. However, the diverse roles of EphA2 and ephrin-A5 in different populations of lens epithelial cells and the unique subcellular localization of these proteins in the lens suggest that they are not a receptor–ligand pair in most cells of the lens [32]. Thus, it is likely that EphA2 and ephrin-A5 interact with other ephrin ligands and Eph receptors, respectively, to regulate the homeostasis of lens epithelial cells and fiber cells.

In this study, we conducted a comprehensive analysis to determine which Eph receptors and ligands are present in adult mouse lenses using RNA isolation, reverse transcription (RT), polymerase chain reaction (PCR), and Sanger sequencing. In addition to screening RNA samples from whole lenses, we separated lens epithelial cells and fiber cells for RNA isolation using our new protocol [38]. We compared 6-week-old samples from control, *EphA2^−/−^*, and *ephrin-A5^−/−^* lenses. Of the 14 Eph receptors and 8 ephrin ligands, we verified that transcripts for 12 Ephs and 8 ephrins are present in the lens. Our data revealed 1 Eph receptor and 1 ephrin ligand that are only expressed in lens epithelial cells. In addition, there is 1 Eph receptor that is expressed in KO lens fiber cells, but not in the control samples. We compared our RT-PCR and sequencing data with microarray data. Overall, the adult mouse lens expresses most EphAs and all EphBs, ephrin-As, and ephrin-Bs; thus, much more work needs to be conducted to identify all cogent receptor–ligand pairs and understand their role in maintaining lens health and homeostasis.

## 2. Materials and Methods

### 2.1. Mice

Mice were maintained in accordance with an approved animal protocol (Indiana University Bloomington Institutional Animal Care and Use Committee, protocol #21-010) and the ARVO Statement for the Use of Animals in Ophthalmic and Vision Research. Generations of *ephrin-A5^−/−^* and *EphA2^−/−^* mice were previously described [31,36,39,40]. All mice were maintained in the C57BL/6J background with wild-type *Bfsp*2 (CP49) genes. Genotyping was completed using automated qPCR on toe and/or tail snips (Transnetyx, Cordova, TN, USA). Male and female littermates were used for experiments.

### 2.2. RNA Isolation from Epithelial Cells

RNA from epithelial cells were obtained by decapsulating freshly dissected lenses using a modified version of a published protocol [38]. Samples were collected from three control (*EphA2^+/+^* and *ephrin-A5^+/+^*) and three knockout (*EphA2^−/−^* and *ephrin-A5^−/−^*) 6-week-old mice. After dissected lenses were cleaned of unrelated tissues, tweezers were used to gently puncture the lens at the equator. Shallow punctures prevented the fiber cells from adhering to the lens capsule and lens epithelial cell layer. A pair of lens capsules with the lens epithelial cells from one mouse were then placed into 0.4 mL of cold TRIzol (Invitrogen, Waltham, MA, USA, Cat# 15596026). Samples were then incubated at room temperature for 30 min. For phase separation, 0.2 mL of chloroform was added to each sample before tubes were shaken vigorously for 15 s. Samples were incubated at room temperature for 10–15 min and centrifuged at 14,000× *g* for 15 min at 4 °C. The aqueous phase was transferred to new RNase-free microcentrifuge tubes. Two volumes of RNA binding buffer per 1 volume of aqueous phase was added to each sample. Then, an equal volume of 100% ethanol (Fisher Scientific, Waltham, MA, USA, Cat# BP2818500), relative to the volume within the tube, was added before the samples were inverted to gently mix. The rest of the RNA isolation was performed with the RNA clean and concentrator kit (Zymo Research, Tustin, CA, USA, Cat# R1013), according to manufacturer instructions and two additional steps. The first additional step was another centrifugation after the final RNA wash buffer centrifugation to remove excess wash buffer. The second additional step was a 2-min waiting step after the addition of DNase/Rnase-free water to the filter in the spin column. The addition of this step allowed for higher recovery of RNA. RNA samples were then incubated at 60 °C for 10 min before being stored at 4 °C overnight for concentration quantification the next day using the NanoDrop One (Thermo Fisher Scientific, Waltham, MA, USA, Version 2.6.0.6., Cat# ND-ONE-W). RNA was stored at −80 °C until use.

### 2.3. RNA Isolation from Fiber Cells, Whole Lens Samples, or Positive Control Samples

RNA isolation from fiber cells, whole lens, or positive control samples were processed using the same protocol [41]. Whole lens and fiber cell samples were collected from four control (*EphA2^+/+^* and *ephrin-A5^+/+^*) and four knockout (*EphA2^−/−^* and *ephrin-A5^−/−^*) 6-week-old mice. Fiber cell samples were collected from the same mice used for epithelial cell RNA isolation, while whole lens RNA sample was isolated from another mouse. A control brain sample was collected from a 9-week-old wild-type C57BL/6J mouse. Lenses from the same mouse were cleaned of other attached tissues, and pairs of lenses or fiber cell masses (after lens capsule and epithelial cell removal) from one mouse were pooled into one sample. For whole lenses and fiber cells masses, 0.4 mL TRIzol was used for homogenization. For the brain positive control sample, 1 mL of TRIzol per 50–100 mg of tissue was used for homogenization. Samples were homogenized with RNase Zap (Sigma-Aldrich, St. Louis, MO, USA, Cat# R2020)-treated polypropylene microcentrifuge pestles, until no large pieces of tissue remained. Homogenized samples were incubated at room temperature for 5 min before 0.4 mL chloroform (Fisher Scientific, Cat# AA22920K2), per 1 mL TRIzol, was added. Samples were then shaken vigorously for 15 s and incubated at room temperature for 3 min. After incubation, the samples were centrifuged at 12,000× *g* for 15 min at 4 °C. The aqueous phase was transferred to RNase-free microcentrifuge tubes. Then, half the sample’s volume of 100% isopropanol (Fisher Scientific, Cat# AC327270010) was added before the samples were mixed by brief vortexing. The samples were incubated at room temperature for 10 min and then centrifuged at 12,000× *g* for 10 min at 4 °C. The supernatant was decanted, and the RNA pellet was washed with 75% ethanol (in diethyl pyrocarbonate (DEPC)-treated water), using a volume equal to the volume of TRIzol used. The samples were then vortexed briefly, so that the pellet floats in solution. Then, the samples were centrifuged at 7500× *g* for 5 min at 4 °C. After centrifugation, the ethanol wash was decanted, and the RNA pellet was allowed to air dry for 5–10 min, with the microcentrifuge tube being inverted. The RNA pellets were then dissolved in 20 μL of DNase/RNase-free water before incubating for 10 min at 60 °C. The samples were then kept at 4 °C overnight for quantification the next day using the NanoDrop One. RNA was stored at −80 °C until use.

### 2.4. Primer Design

Primer design was performed with the National Center for Biotechnology Information (NCBI) Primer-BLAST website [https://www.ncbi.nlm.nih.gov/tools/primer-blast/ (accessed on 2 September 2022)]. Primers were designed to span exon–exon junctions, when possible, and were specific to each gene. For validated variants, specific primer sets were designed for each variant, when possible, utilizing the differences in sizes of the resulting PCR products from one set of primers or sequencing when the difference is <15 nucleotides. PCR products were between 500–2200 bp (Table S1).

### 2.5. Reverse Transcription and Polymerase Chain Reaction (RT-PCR)

Reverse transcription was performed using SuperScript III (Thermo Fisher Scientific, Cat.# 18080-051) and Oligo(dT)_20_ primer (50 µM), following the manufacturer’s instructions. The cDNA was used immediately or stored at −20 °C until use.

Polymerase chain reaction was performed using Quick-Load *Taq* 2X master mix (New England Biolabs, Ipswich, MA, USA, Cat# M0271S), following the manufacturer instructions. The reactions were loaded into the MiniAmp Thermal Cycler (Thermo Fisher Scientific, Version 0.2.9, Cat# A37834). The thermocycling conditions are as follows. There was one cycle of 95 °C for 30 s, followed by 45 cycles of 95 °C for 30 s, 53.5–55 °C for 30 s (temperature varied, based on the primers, info provided in Table S1) and 68 °C for 1 min per 1 kb of expected PCR product length. A final cycle of 68 °C for 5 min finished the PCR reaction. PCR products were maintained at 4 °C before storage at −20 °C or gel electrophoresis.

### 2.6. Gel Electrophoresis and DNA Extraction from Gel Pieces

Gel electrophoresis was performed using 0.8% or 2% agarose (Fisher Scientific, Cat# S53) gels with GelGreen (Biotium, Fremont, CA, USA, Cat# 41005). The DNA ladder used was GeneRuler 100 bp (Thermo Fisher Scientific, Cat# SM0241) or GeneRuler 1 kb Plus (Thermo Fisher Scientific, Cat# SM1331). Samples with multiple variants and with product sizes under 1000 bp were run on 2% gels to better separate the bands for gel extraction and DNA sequencing. Gels were imaged using PrepOne Sapphire Blue LED illuminator (Embi Tec, San Diego, CA, USA, Cat# PI-1000). Gel extraction was performed by cutting out the individual bands from the gels using a clean razor and placing the gel piece into a microcentrifuge tube that was then processed with the QIAquick gel extraction kit (Qiagen, Hilden, Germany, Cat# 28704), following the manufacturer’s instructions. The extracted DNA was then prepared for sequencing, according to Quintarabio’s (Cambridge, MA, USA) sample submission and shipping instructions. The sequencing results were compared in NCBI nucleotide BLAST to confirm the identity of each PCR product.

### 2.7. Microarray Data Comparison

Microarray data were obtained from the iSyTE database [https://research.bioinformatics.udel.edu/iSyTE/ppi/expression.php (accessed on 13 September 2022)], searching for Ephs and ephrins. A standard lens gene expression search was performed for mouse mm10 species, with normalized expression comparison. The data that were used for Table 2 and Table S2 were from the developmental dataset for Affymetrix 430 2.0 epithelial P28 and P56, as well as Illumina WG-6 v2.0 P30, P42, and P52. For Table 2, we also listed other mouse tissues with high expression of Ephs and ephrins, according to each gene’s expression level information in the NCBI gene database.

## 3. Results

### 3.1. EphA Transcripts in the Lens

**Figure 1:**
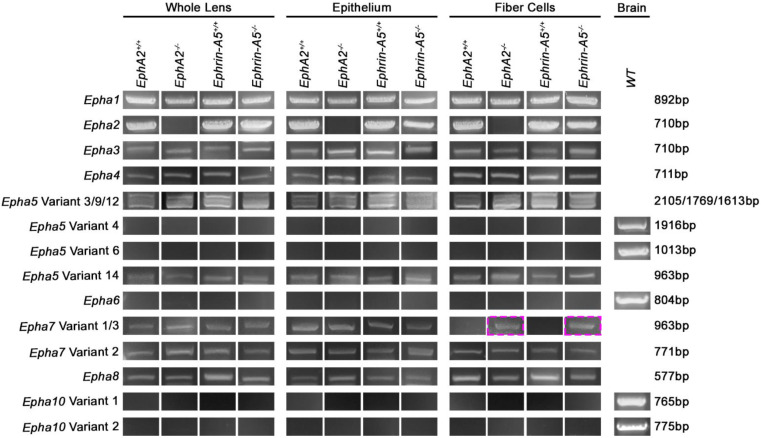
*Epha* transcripts in whole lens, lens epithelial cell, or lens fiber cell RNA samples from 6-week-old *EphA2^+/+^*, *EphA2^−/−^*, *ephrin-A5^+/+^*, and *ephrin-A5^−/−^* mice. Positive control was brain RNA isolated from a wild-type (*WT*) control mouse. We detected *Epha1*, *Epha2*, *Epha3* variant 1, *Epha4*, *Epha5* variants 3, 9, 12, and 14, *Epha7* variants 1 and 2, and *Epha8* in the lens. As expected, *Epha2* was not detected in *EphA2^−/−^* lens samples. *Epha7* variant 1 was detected in all whole lens and epithelial cell samples and in the fiber cells of *EphA2^−/−^* and *ephrin-A5^−/−^* lenses (magenta boxes). We did not detect *Epha6* or *Epha10* in the lens.

We analyzed RNA transcripts with specific primer pairs for *Epha1–8* and *Epha10* in control, *EphA2^−/−^*, and *ephrin-A5^−/−^* whole lens, epithelial cell, and fiber cell samples from 6-week-old mice. We found transcripts for *Epha1*, *Epha2*, *Epha3* variant 1, *Epha4*, *Epha5* variants 3, 9, 12, and 14, *Epha7* variant 2, and *Epha8* in all samples tested (Figure 1). As expected, *Epha2* transcripts are absent from the *EphA2^−/−^* samples. Variants 1 and 2 of *Epha3* differ by one amino acid, and *Epha3* variant 1 has an additional amino acid, Q478. The presence of *Epha3* variant 1 in lens samples was confirmed by sequencing of the PCR product. *Epha5* has 14 different variants with different splicing patterns and lengths. We used sequencing to confirm the presence of four variants of *Epha5* in the lens. *Epha7* has three variants, 1, 2, and 3. *Epha7* variants 1 and 3 differ by four amino acids, and variant 1 is longer, with the addition of 601–604 KFPG amino acids. We detected *Epha7* variant 1 in whole lens and epithelial cells samples of the control and KO samples, as well as in KO fiber cell samples (Figure 1, magenta boxes). *Epha7* variant 2 is a shorter variant lacking multiple exons and has a different and shorter C-terminus than variants 1 and 3. Sequencing confirmed the presence of the *Epha7* variant 2 in all lens samples. We did not detect *Epha6* or *Epha10* in any samples. There are two variants, 1 and 2, for *Epha10*. Variant 2 of *Epha10* is much shorter than variant 1, due to loss of exons, and has a unique C-terminus, compared to variant 1. Positive control PCR products from brain RNA samples were used to confirm primer sets and PCR conditions for the *Epha*5 variants 1, 4, 5, 6, 8, and 10 and the *Epha6* and *Epha10* experiments.

### 3.2. EphB Transcripts in the Lens

**Figure 2:**
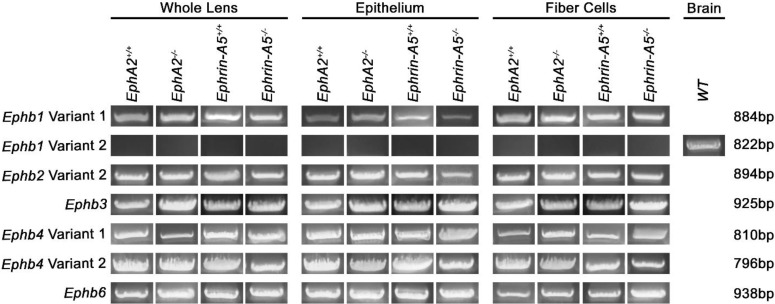
*Ephb* transcripts in whole lens, lens epithelial cell, or lens fiber cell RNA samples from 6-week-old *EphA2^+/+^*, *EphA2^−/−^*, *ephrin-A5^+/+^*, and *ephrin-A5^−/−^* mice. Positive control was brain RNA isolated from a *WT* control mouse. We detected *Ephb1* variant 1, *Ephb2* variant 2, *Ephb3*, *Ephb4* variants 1 and 2, and *Ephb6* in the lens. We did not detect *Ephb1* variant 2 in the lens.

Next, we tested for the presence of *Ephb1–4* and *Ephb6* transcripts in the control and KO samples. We detected *Ephb1* variant 1, *Ephb2* variant 2, *Ephb3*, *Ephb4* variants 1 and 2, and *Ephb6* transcripts in all lens samples (Figure 2). The *Ephb1* variant 1 is a longer isoform, and variant 2 lacks one of the coding exons. *Ephb1* variants 1 and 2 have the same N- and C-termini. Specific primers were designed to distinguish between *Ephb1* variants 1 and 2. The lens only expresses *Ephb1* variant 1, and the primers for *Ephb1* variant 2 were verified by the positive control brain RNA sample. *Ephb2* variant 1 has one additional amino acid, Q477, compared to variant 2. Sequencing verified that the lens expresses *Ephb2* variant 2. *Ephb4* also has two variants, and due to an alternate in-frame splice site, variant 2 is a shorter transcript. Specific primers designed for each variant and sequencing confirm that both variants 1 and 2 of *Ephb4* are expressed in the lens. *Ephb6* also has two variants, but the two variants are identical in the coding region and differ by a four-nucleotide difference in the 5′ untranslated region (UTR). 

In total, the lens expresses 12 of the 14 *Eph* genes. Due to the variants in multiple genes, we found 14 *Eph* transcripts in our samples. Most notably, the *Epha7* variant 1 is normally only expressed in lens epithelial cells, but is present in the lens fiber cells of *EphA2^−/−^* and *ephrin-A5^−/−^* samples.

### 3.3. Ephrin-A Transcripts in the Lens

**Figure 3:**
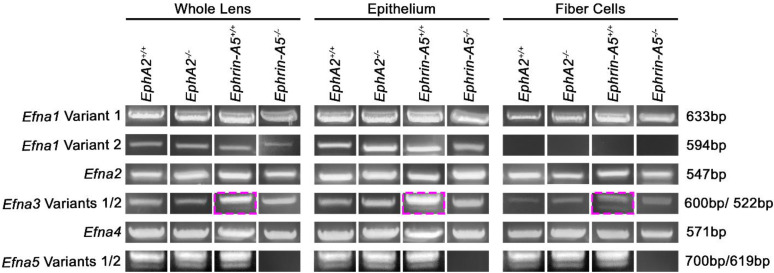
*Efna* transcripts, which encode for ephrin-A proteins, in whole lens, lens epithelial cell, or lens fiber cell RNA samples from 6-week-old *EphA2^+/+^*, *EphA2^−/−^*, *ephrin-A5^+/+^*, and *ephrin-A5^−/−^* mice. We detected *Efna1* variant 1, *Efna2*, *Efna3* variant 1, *Efna4*, and *Efna5* variants 1 and 2 in the lens. *Efna1* variant 2 was detected in the whole lens and epithelial cells, but not detected in fiber cells. *Efna3* variant 2 was only detected in *ephrin-A5^+/+^* lens samples (magenta boxes).

Ephrin-A proteins are encoded by the *Efna* genes. We performed experiments to determine whether *Efna1–5* are present in the lens. Our results show that *Efna1* variant 1, *Efna2*, *Efna3* variant 1, *Efna4*, and *Efna5* variants 1 and 2 transcripts are present in all lens samples (Figure 3). As expected, the *Efna5* transcripts are absent in the *ephrin-A5^−/−^* samples. *Efna1* variant 2 is missing a part of the 5′ UTR and coding region, resulting in a shorter transcript, compared to variant 1. Specific forward primers for *Efna1* variants 1 and 2 revealed that the lens expresses the longer *Efna1* variant 1 transcript in both epithelial cells and fiber cells, while *Efna1* variant 2 is only expressed in lens epithelial cells. *Efna3* has 7 variants. *Efna3* variants 1 and 2 differ by one exon in the 3′ coding region and can be distinguished by PCR product size. We found *Efna3* variant 1 transcripts in all lens samples. With sequencing verification, we could only detect very low levels of *Efna3* variant 2 transcripts in the *ephrin-A5^+/+^* control samples (Figure 3, magenta boxes). *Efna3* variants 3, 4, and 5 have identical coding regions and are shorter transcripts with a start codon in the middle of the variant 1 sequence. There are minor differences in the 5′ UTR of *Efna3* variants 3, 4, and 5 from the 5′ coding region of *Efna3* variant 1; thus, specific primers to test for *Efna3* variants 3, 4, and 5 could not be designed. *Efna3* variants 6 and 7 have identical coding regions and are shorter transcripts with a start codon in the middle of the variant 2 sequence. There are some differences in the 5′ UTR of variants 6 and 7 from the 5′ coding region of variant 2, but specific primers could not be designed to distinguish between variants 2, 6, and 7. Interestingly, the lens expresses both variants of *Efna5*. *Efna5* variant 2 lacks one exon and is shorter than variant 1, but both transcripts have the same N- and C-termini sequence.

### 3.4. Ephrin-Bs Transcripts in the Lens

**Figure 4:**
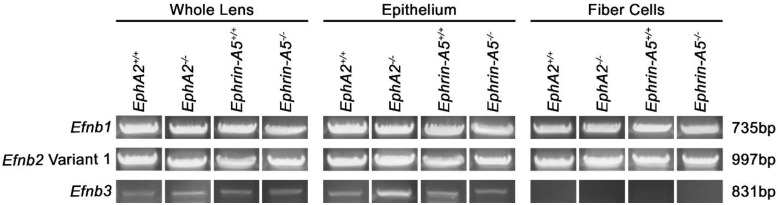
*Efnb* transcripts, which encode for ephrin-B proteins, in whole lens, lens epithelial cell, or lens fiber cell RNA samples from 6-week-old *EphA2^+/+^*, *EphA2^−/−^*, *ephrin-A5^+/+^*, and *ephrin-A5^−/−^* mice. We detected *Efnb1* and *Efnb2* variant 1 in the lens. *Efnb3* was detected in the whole lens and epithelial cells, but not detected in fiber cells.

The last group of genes tested were *Efnb1–3*, which encode for ephrin-B1–3. We detected *Efnb1* and *Efnb2* variant 1 in all lens samples (Figure 4). *Efnb2* has two variants, and variant 2 is shorter by 93 base pairs. *Efnb2* variants 1 and 2 have the same N- and C-termini sequence. We did not detect the smaller *Efnb2* variant 2 band in any of the lens samples. Interestingly, *Efnb3* transcripts are only found in lens epithelial cells. Overall, we found transcripts for all eight *Efn* genes in the lens. Notably, *Efna5* variants 1 and 2 were both expressed in the lens, and *Efnb3* was only found in lens epithelial cells. All PCR and sequencing results are summarized in Table 1.

### 3.5. Data Comparison with Adult Lens Microarray Data

We compared our RT-PCR results to the *Eph* and *Efn* microarray data available in iSyte 2.0 from the Affymetrix 430 2.0 and Illumina WG-6 v2.0 arrays [42]. We chose to compare our data from 6-week-old mice with data from wild-type lenses at postnatal day 28 (P28) from the epithelium and at P56 from the whole lens on the Affymetrix platform and results from wild-type whole lenses from P30, P42, and P52 on the Illumina arrays with our data. It should be noted that the data from the two different microarray platforms should not be compared to each other, due to differences in technology for the two arrays. We designated detected (✓) or not detected (n.d.) for the microarray data (Table 2) and included the normalized lens expression numbers for each gene from iSyte 2.0 (Supplemental Table S2). The normalized lens expression values for the two different chip sets were in different ranges. Our data matches closely with the microarray data, except for a few of the genes. We detected the expression of *Epha8*, but the array data did not, and we did not detect *Epha6*, but the Affymetrix array did. Neither microarray tested for *Epha10*.

In Table 2, we also list other mouse tissues with high expression of each *Eph* or *Efn*. We wanted to determine whether there were other tissues with similar expression patterns of *Eph* or *Efn* as in the lens. The lens expresses many of the genes also detected in the brain, lung, and the gastrointestinal tract. However, no other tissues expressed as many *Eph* or *Efn*, compared to the lens. 

## 4. Discussion

Our data shows that the adult mouse lens expresses 7 *Epha*, 5 *Ephb*, 5 *Efna*, and 3 *Efnb* transcripts. Counting all the different variants, we detected 18 *Ephs* and 11 *Efns* in the lens. Of these isoforms, three are only expressed in lens epithelial cells, *Epha7* variant 1, *Efna1* variant 2, and *Efnb3*. *Epha7* variant 1 is also aberrantly expressed in the *EphA2^−/−^* and *ephrin-A5^−/−^* lens fiber cells. Interestingly, we only found *Efna3* variant 2 in the control *ephrin-A5^+/+^* samples. This variant has very faint PCR bands, but was consistently present in the control *ephrin-A5^+/+^* whole lens, epithelial cell, and fiber cell samples. It is not clear why *EphA2^+/+^* samples do not also express *Efna3* variant 2. However, this does suggest that, even among control “wild-type” animals, there could be slight strain differences. This notion is supported by our previous work, showing differences between our control animals vs. pure C57BL6/J wild-type mice [43].

Our data reveals that the loss of EphA2 or ephrin-A5 causes abnormal expression of *Epha7* variant 1 transcripts in KO lens fiber cells. This unexpected result was our first clue about compensatory upregulation or deregulation in KO animals. In addition to our data, a recent work described marked downregulation of *Epha5* transcripts in developing lenses with the disruption of the musculoaponeurotic fibrosarcoma (*MAF*) family of proteins [44]. The data from RNA-seq plates did not distinguish between the 14 variants of *Epha5*. MAFs encode basic leucine zipper transcription factors that are known to be important for lens development and to be involved cataractogenesis [44,45,46,47,48,49,50]. There may be other insights that can be gleaned from the microarray data between the control, *EphA2^−/−^*, and *ephrin-A5^−/−^* lens samples. Comparison of our RT-PCR results and previous microarray data indicate a good match between the information from these data sets for most of the *Eph* and *Efn* isoforms. We may be able to use microarrays to quickly screen our KO lenses for highly upregulated or downregulated *Ephs* and *Efns* and examine large groups of other genes.

Our new method to isolate RNA from epithelial cells allows for ~20–30 PCR reactions, allowing for the efficient screening of transcripts. This protocol makes it possible to identify epithelial-cell specific isoforms. We had presumed that, in the whole lens samples, the fiber cell RNA content would dominate over the smaller amount of RNA from the lens epithelial cells. Different from our investigation of tropomyosin transcripts in whole lens [41] vs. epithelial cells [51], where several isoforms of tropomyosin were only detected in epithelial cell samples, we surprisingly found three epithelial cell-specific *Eph*/*Efn* transcripts (*Epha7* variant 1, *Efna1* variant 2, and *Efnb3*) that were also present in whole lens samples. Of note, *Efnb3* did not have other variants expressed in fiber cells, but it was detected on both microarray platforms in both the epithelial and whole lens samples. Our data suggests that RNA from epithelial cells can be detected in whole lens samples; however, very low expression epithelial-cell-exclusive genes may still be difficult to detect in whole lens samples.

The large number of isoforms and variants of *Ephs* and *Efns* found in adult mouse lenses greatly complicates our search for lens receptor–ligand pairs. EphA receptors mainly bind to ephrin-As, while EphB receptors usually interact with ephrin-Bs [52,53]. Less common are the cross-interactions between EphAs and ephrin-Bs or EphBs and ephrin-As [27,54]. Each receptor can interact with multiple ligands, and similarly, each ligand can bind to multiple receptors [54]. This leads to a complex and large matrix of possible receptor–ligand pairs. To tackle the next phase of this project, we will be designing specific TaqMan real-time PCR assays to uncover changes in *Eph* and *Efn* expression levels between our control and KO samples. This may help us narrow down the list of priority isoforms to study in our KO mouse lines. Following the identification of priority isoforms for study, we plan to perform Western blotting, co-immunoprecipitation, immunostaining, and/or proximity ligation assay to determine the protein localization and receptor–ligand pairs.

## Figures and Tables

**Table 1:** *Eph* and *Efn* transcripts in the lens.

Gene	Whole Lens				Epithelium				Fiber Cells			
	*EphA2*		*Ephrin-A5*		*EphA2*		*Ephrin-A5*		*EphA2*		*Ephrin-A5*	
	*+/+*	*-/-*	*+/+*	*-/-*	*+/+*	*-/-*	*+/+*	*-/-*	*+/+*	*-/-*	*+/+*	*-/-*
***Epha1***	**✓**				**✓**				✓			
***Epha2***	**✓**	KO	**✓**	**✓**	**✓**	KO	**✓**	**✓**	**✓**	KO	**✓**	**✓**
***Epha3* Variant 1**	**✓**				**✓**				**✓**			
*Epha3* Variant 2	n.d.				n.d.				n.d.			
***Epha4***	**✓**				**✓**				**✓**			
*Epha5* Variant 1	n.d.				n.d.				n.d.			
*Epha5* Variant 2	n.d.				n.d.				n.d.			
***Epha5 * Variant 3**	**✓**				**✓**				**✓**			
*Epha5* Variant 4	n.d.				n.d.				n.d.			
*Epha5* Variant 5	n.d.				n.d.				n.d.			
*Epha5* Variant 6	n.d.				n.d.				n.d.			
*Epha5* Variant 7	n.d.				n.d.				n.d.			
*Epha5* Variant 8	n.d.				n.d.				n.d.			
***Epha5 * Variant 9**	**✓**				**✓**				**✓**			
*Epha5* Variant 10	n.d.				n.d.				n.d.			
*Epha5* Variant 11	n.d.				n.d.				n.d.			
***Epha5* Variant 12**	**✓**				**✓**				**✓**			
*Epha5* Variant 13	n.d.				n.d.				n.d.			
***Epha5 * Variant 14**	**✓**				**✓**				**✓**			
*Epha6*	n.d.				n.d.				n.d.			
***Epha7 * Variant 1**	**✓**				**✓**				n.d.	**✓**	n.d.	**✓**
***Epha7 * Variant 2**	**✓**				**✓**				**✓**			
*Epha7* Variant 3	n.d.				n.d.				n.d.			
***Epha8***	**✓**				**✓**				**✓**			
*Epha10* Variant 1	n.d.				n.d.				n.d.			
*Epha10* Variant 2	n.d.				n.d.				n.d.			
***Ephb1 * Variant 1**	**✓**				**✓**				**✓**			
*Ephb1* Variant 2	n.d.				n.d.				n.d.			
*Ephb2* Variant 1	n.d.				n.d.				n.d.			
***Ephb2 * Variant 2**	**✓**				**✓**				**✓**			
***Ephb3***	**✓**				**✓**				**✓**			
***Ephb4 * Variant 1**	**✓**				**✓**				**✓**			
***Ephb4 * Variant 2**	**✓**				**✓**				**✓**			
***Ephb6* Variant 1**	**✓**				**✓**				**✓**			
***Ephb6* Variant 2**	**✓**				**✓**				**✓**			
***Efna1 * Variant 1**	**✓**				**✓**				**✓**			
***Efna1* Variant 2**	**✓**				**✓**				n.d.			
***Efna2***	**✓**				**✓**				**✓**			
*Efna3 * Variant 1 *	**✓**				**✓**				**✓**			
*Efna3 * Variant 2 *	n.d.	n.d.	**✓**	n.d.	n.d.	n.d.	**✓**	n.d.	n.d.	n.d.	**✓**	n.d.
***Efna4***	**✓**				**✓**				**✓**			
***Efna5* Variant 1**	**✓**	**✓**	**✓**	KO	**✓**	**✓**	**✓**	KO	**✓**	**✓**	**✓**	KO
***Efna5 * Variant 2**	**✓**	**✓**	**✓**	KO	**✓**	**✓**	**✓**	KO	**✓**	**✓**	**✓**	KO
***Efnb1***	**✓**				**✓**				**✓**			
***Efnb2 * Variant 1**	**✓**				**✓**				**✓**			
*Efnb2* Variant 2	n.d.				n.d.				n.d.			
***Efnb3***	**✓**				**✓**				n.d.			

*+/+* = wild-type or control; *−/−* or KO = knockout; n.d. = not detected; blue = detected in all lens fractions; orange = detected in some lens fractions; yellow highlight = detected in lens epithelial fractions, but not in lens fiber cell fractions; * There are two shorter variants of *Efna3*, variants 3/4/5 and variants 6/7, that cannot be distinguished by RT-PCR.

**Table 2:** *Eph* and *Efn* transcripts in lens microarray studies and other tissues.

Gene	iSyte 2.0					NCBI
	Affymetrix 430 2.0		Illumina WG-6 v2.0			
	P28 Epi	P56	P30	P42	P52	Other tissues with high expression
***Epha1***	**✓**	**✓**	**✓**	**✓**	**✓**	Duodenum, intestines, lung
***Epha2***	**✓**	**✓**	**✓**	**✓**	**✓**	Duodenum, intestines, lung
***Epha3***	**✓**	**✓**	**✓**	**✓**	**✓**	Embryonic/adult brain, embryonic limb
***Epha4***	**✓**	**✓**	**✓**	**✓**	**✓**	Embryonic/adult brain, embryonic limb, heart
***Epha5***	**✓**	**✓**	**✓**	**✓**	**✓**	Embryonic/adult brain
*Epha6*	**✓**	**✓**	n.d.	n.d.	n.d.	Adult brain
***Epha7***	**✓**	**✓**	**✓**	**✓**	**✓**	Embryonic/adult brain, embryonic limb
***Epha8 ^1^***	n.d	n.d	n.d	n.d	n.d	Embryonic brain, adult cerebellum
*Epha10 ^2^*	N/A	N/A	N/A	N/A	N/A	Embryonic/adult brain, testis
***Ephb1***	**✓**	**✓**	**✓**	**✓**	**✓**	Embryonic/adult brain
***Ephb2***	**✓**	**✓**	**✓**	**✓**	**✓**	Embryonic brain, adrenal, colon, intestines
***Ephb3***	**✓**	**✓**	n.d.	n.d.	n.d.	Embryonic limb, colon, lung, stomach
***Ephb4***	**✓**	**✓**	**✓**	**✓**	**✓**	Embryonic limb, colon, lung, ovary
***Ephb6***	**✓**	**✓**	**✓**	**✓**	**✓**	Thymus, adult cortex
***Efna1***	**✓**	**✓**	**✓**	**✓**	**✓**	Duodenum, lung, intestines
***Efna2***	**✓**	**✓**	n.d.	n.d.	n.d.	Embryonic brain, embryonic liver, ovary
***Efna3***	**✓**	**✓**	n.d.	n.d.	n.d.	Embryonic brain, embryonic limb, stomach
***Efna4***	**✓**	**✓**	**✓**	**✓**	**✓**	Embryonic limb, duodenum, ovary
***Efna5***	**✓**	**✓**	**✓**	**✓**	**✓**	Embryonic brain, embryonic limb, bladder
***Efnb1***	**✓**	**✓**	**✓**	**✓**	**✓**	Colon, duodenum, lung, ovary
***Efnb2***	**✓**	**✓**	**✓**	**✓**	**✓**	Colon, lung
***Efnb3 ^3^***	**✓**	**✓**	**✓**	**✓**	**✓**	Embryonic brain, embryonic limb, heart

n.d. = not detected. Bolded genes were detected in our RT-PCR experiments; ^1^ *Epha8* was detected at low levels in E15.5 and P0 lenses on the Illumina WG-6 v1.1 microarray; ^2^ *Epha10* was not tested in any of the reported arrays on iStye 2.0; ^3^ *Efnb3 i*s only expressed in lens epithelial cells.

## Data Availability

The authors will provide a detailed description of methods and original data upon request.

## Supplementary Materials

The following supporting information can be downloaded at: https://www.mdpi.com/article/10.3390/cells11203291/s1, Table S1: Primers, PCR product size, and PCR temperature; Table S2: Normalized lens expression of Eph and Efn transcripts in lens microarray studies.
