# Supplementary material for: Mapping the Universe of Eph Receptor and Ephrin Ligand Transcripts in Epithelial and Fiber Cells of the Eye Lens
Source: Cells. 2022 Oct 19;11(20):3291. doi: 10.3390/cells11203291 (PMC9600312; doi:10.3390/cells11203291)
Supplement: Supplementary file 1 [file cells-11-03291-s001.zip › cells-1952310-supplementary/Supplemental Table S1.pdf]

Supplemental Table S1: Primers, PCR product size, and PCR temperature

| Gene (Variant) | Ascension Number | Forward Primer | Reverse Primer | PCR Product(s) | PCR Temp |
|---|---|---|---|---|---|
| *Epha1* | NM_023580.4 | AGCTTCTCCACATCAGGGAC | CACCAAACTCCCCTTCTCCTA | 892bp | 54°C |
| *Epha2* | NM_010139.3 | GTCCATGTCTTGCACACGTC | CAACTCCTGCCAGTACCAGA | 701bp | 54°C |
| *Epha3* (1/2)[1] | NM_010140.4/ NM_001362452.1 | CATGGCTTGTACCCGACCTC | TGACCACCGTGAGGACAATG | 722bp | 55°C |
| *Epha4* | NM_007936.3 | GAGAGCTTGGGTGGATAGCA | TTTTGCAAGCTTGGCATTCA | 711bp | 53.5°C |
| *Epha5* (1/4/5/8/10) | NM_001402749.1/ NM_001402752.1/ NM_001402753.1/ NM_007937.3/ NM_001402757.1 | TGGGAAATGCATGTGCAAGG | GCACCATCCCGTTTACCATC | 2186bp/ 1916bp/ 1850bp/ 1760bp/ 1694bp | 55°C |
| *Epha5* (2/3/7/9/11/ 12/13)[2] | NM_001402750.1/ NM_001402751.1/ NM_001402755.1/ NM_001402756.1/ NM_001402758.1/ NM_001402759.1/ NM_001402760.1 | CTCTCTGGCAGGCTGCTATT | TCAGAAAAACTACTCACTCCAAGGT | 2171bp/ 2105bp/ 1835bp/ 1769bp/ 1679bp/ 1613bp/ 1610bp | 55°C |
| *Epha5* (for sequencing)[3] | N/A | N/A | GGGATCTTCATAAGTGTGCGGA | N/A | N/A |
| *Epha5* (6)[4] | NM_001402754.1 | CTCTCTGGCAGGCTGCTATT | TGACTGGAGAAGGAGCTTGG | 1013bp | 55°C |
| *Epha5* (14) | NM_001402761.1 | CTCTCTGGCAGGCTGCTATT | CTCACACAAGTTTGCCTGGAA | 650bp | 55°C |
| *Epha6* | NM_007938.2 | GACAGGCTACAGTGGCTACA | TACTTCATGCCTGACGCGAT | 804bp | 55°C |
| *Epha7* (1/3)[5] | NM_010141.4/ NM_001290434.1 | AGCAAGGCTGACCAAGAAGG | AGTTCCCAGGGGTGTTTTCA | 963bp | 55°C |

| Gene (Variant) | Ascension Number | Forward Primer | Reverse Primer | PCR Product(s) | PCR Temp |
|---|---|---|---|---|---|
| *Epha7* (2)[6] | NM_001122889.1 | ACAACGGGGGAAGAAACGAT | CGGTTTATAAAACTGACAGGTGCT | 771bp | 55°C |
| *Epha8* | NM_007939.2 | GAAGATCATAGGCTCGGGGGA | CTCCCACATGACCACACCAA | 577bp | 55°C |
| *Epha10* (1)[7] | NM_001256432.2 | TATCCTCCTGGACTCCAAAGC | TTGTAAAACCCTGGGGGACA | 765bp | 55°C |
| *Epha10* (2)[7] | NM_177671.6 | TATCCTCCTGGACTCCAAAGC | TCCCTATCCCATCCCTCTGG | 775bp | 55°C |
| *Ephb1* (1)[7] | NM_173447.3 | GAGGTGGCGTGTACTAGTGT | AATTCCCGGACGGCTTCATT | 884bp | 55°C |
| *Ephb1* (2)[7] | NM_001168296.1 | GAGGTGGCGTGTACTAGTGT | GAACTCCCCTCGGCCTGT | 822bp | 55°C |
| *Ephb2* (1/2)[8] | NM_001290753.2/ NM_010142.4 | TCAGGAACCTTCAAGGCCAA | CTCAAACCCCCGTCTGTTAC | 894bp | 55°C |
| *Ephb3* | NM_010143.1 | CGGACGAGATGACCTCCTTT | GTATCCCACCTTCAGCGTCT | 925bp | 55°C |
| *Ephb4* (1)[7] | NM_001159571.1 | AGCAAAGCTGGCTTCTACCT | AGAAAGAAACCCAATGCGCC | 810bp | 55°C |
| *Ephb4* (2)[7] | NM_010144.6 | AGCAAAGCTGGCTTCTACCT | GAGGCACCTCACGGTCA | 796bp | 55°C |
| *Ephb6* (1/2)[9] | NM_007680.5/ NM_001146351.2 | CCGAGAGACCTTCACCCTTT | CTCGACTCTCAGTCAGTCCC | 938bp | 55°C |
| *Efna1* (1)[10] | NM_010107.4 | GCGCTATGGAGTTCCTTTGG | GCGTACACCCTCACTGAGAT | 633bp | 55°C |
| *Efna1* (2)[10] | NM_001162425.1 | GCTGATTCGAGAGGTTCCGT | GCGTACACCCTCACTGAGAT | 549bp | 55°C |

| Gene (Variant) | Ascension Number | Forward Primer | Reverse Primer | PCR Product(s) | PCR Temp |
|---|---|---|---|---|---|
| *Efna2* | NM_007909.3 | GCTGACCGATACGCAGTCTA | GACACTAGGAGCCCAGAAGG | 547bp | 55°C |
| *Efna3* (1/2)[11] | NM_010108.2/ NM_001377116.1 | CGGCATGCGGTATACTGGAAC | CTAGGAGGCCAAGAGCGTC | 600bp/ 522bp | 55°C |
| *Efna4* | NM_007910.2 | ACTCCTCTAACCCCAGGTTG | ACTCTCAGGAGACGGAGGAT | 517bp | 55°C |
| *Efna5* (1/2)[11] | NM_207654.2/ NM_010109.3 | AGGCGTGATGTTGCACG | ACTGTGCTATAATGTCAAAAGCATC | 700bp/ 619bp | 53°C |
| *Efnb1* | NM_010110.5 | TCACTTGCAACAAGCCACAC | TGTAGTAGATGTTCGCCGGG | 735bp | 55°C |
| *Efnb2* (1/2)[11] | NM_010111.6/ NM_001368299.1 | GAGGGACTCTGTGTGGAAGT | CAGGCCTCAGACCTTGTAGT | 997bp/ 904bp | 55°C |
| *Efnb3* | NM_007911.5 | TCGGCGAATAAGAGGTTCCA | CTTCTCGTAGTGAGGGCAGA | 831bp | 55°C |

[1]Variant 1 has one additional amino acid Q478 due to an AGC insertion at 1558-1560 in the mRNA sequence.
[2]This set of primers will also amplify *Epha5* variant 6 with the same size product as variant 11. Thus, a different set of primers was used to amplify variant 6.
[3]To distinguish the large number of *Epha5* variants, a sequencing primer was designed for the middle of the amplified transcripts to distinguish between variants 1/4/5/8/10 and variants 2/3/7/9/11/12/13.
[4]These primers will also amplify *Epha5* variants 8, 10, 11, 12, and 13 for a product that is 848bp. The brain control and lens samples have a band at 848bp indicating the PCR reaction worked, but no larger band at 1013bp for variant 6 was detected.
[5]Compared to variant 3, variant 1 has four additional amino acids KFPG (601-604) due to a TTAAATTTCCAG insertion at 2052-2063 in the mRNA sequence.
[6]Reverse primer is specific for variant 2.
[7]Reverse primers are specific for each variant.
[8]Variant 1 has one additional amino acid Q477 due to an AGC insertion at 1609-1611 in the mRNA sequence.
[9]Variants 1 and 2 are identical in the coding region.
[10]Forward primers are specific for each variant.
[11]Primers amplify two variants that can be distinguished by size.
