# Supplementary material for: Mapping the Universe of Eph Receptor and Ephrin Ligand Transcripts in Epithelial and Fiber Cells of the Eye Lens
Source: Cells. 2022 Oct 19;11(20):3291. doi: 10.3390/cells11203291 (PMC9600312; doi:10.3390/cells11203291)
Supplement: Supplementary file 1 [file cells-11-03291-s001.zip › cells-1952310-supplementary/Supplemental Table S2.pdf]

Supplemental Table S2: Normalized lens expression of *Eph* and *Efn* transcripts in lens microarray studies

**Gene**	**iSyte 2.0**				
	Affymetrix 430 2.0		Illumina WG-6 v2.0		
	P28 Epi	P56	P30	P42	P52
Epha1	44.79	111.1	120.95	124.05	124.4
Epha2	172.23	253.03	172.3	169.7	158.56
Epha3	16.96	9.63	120.72	124.06	127.83
Epha4	25.89	44.74	136.55	130.21	129.43
Epha5	52.76	45.61	118.82	113.87	115.64
Epha6	10.7	13.13	-	-	-
Epha7	33.93	33.04	121.42	121.17	120.56
Epha8	-	-	-	-	-
Epha10[1]	N/A	N/A	N/A	N/A	N/A
Ephb1	89.62	74.21	122.21	119.17	122.66
Ephb2	87.37	78.92	117.17	117.65	117.57
Ephb3	349.11	120.57	-	-	-
Ephb4	70.36	92.6	239.92	173.28	182.6
Ephb6	43.06	34.7	120.42	119.67	119.57
Efna1	308.08	139.63	149.89	140.32	142.18
Efna2	57.12	55.5	-	-	-
Efna3	49.09	54.84	-	-	-
Efna4	43.75	50.13	121.06	126.32	127.08
Efna5	857.18	284.66	710.8	497.28	508.74
Efnb1	234.14	169.92	187.98	205.52	207.77
Efnb2	1321.02	831.39	157.92	129.08	129.98
Efnb3	86.25	42.78	127.59	123.65	121.87

Bolded genes were detected in our RT-PCR experiments.
[1]*Epha10* was not tested in any of the reported arrays on iStye 2.0.
